# The Microbiota–Gut–Brain Axis in Depression: The Potential Pathophysiological Mechanisms and Microbiota Combined Antidepression Effect

**DOI:** 10.3390/nu14102081

**Published:** 2022-05-16

**Authors:** Fangyuan Zhu, Huaijun Tu, Tingtao Chen

**Affiliations:** 1 Departments of Geriatrics, The Second Affiliated Hospital of Nanchang University, Nanchang 330031, China; zhufy719@163.com; 2 Queen Mary School, Nanchang University, Nanchang 330031, China; 3 National Engineering Research Center for Bioengineering Drugs and Technologies, Institute of Translational Medicine, Nanchang University, Nanchang 330031, China

**Keywords:** depression, probiotics, gut–brain axis, gut microbiota

## Abstract

Depression is a kind of worldwide mental illness with the highest morbidity and disability rate, which is often accompanied by gastrointestinal symptoms. Experiments have demonstrated that the disorder of the intestinal microbial system structure plays a crucial role in depression. The gut–brain axis manifests a potential linkage between the digestion system and the central nervous system (CNS). Nowadays, it has become an emerging trend to treat diseases by targeting intestinal microorganisms (e.g., probiotics) and combining the gut–brain axis mechanism. Combined with the research, we found that the incidence of depression is closely linked to the gut microbiota. Moreover, the transformation of the gut microbiota system structure is considered to have both positive and negative regulatory effects on the development of depression. This article reviewed the mechanism of bidirectional interaction in the gut–brain axis and existing symptom-relieving measures and antidepression treatments related to the gut microbiome.

## 1. Introduction

Depression is described as a state characterized by a low mood and aversion to activity, considered as one of the most prevalent mental and behavioral disorders around the world [1], which is mainly caused by a combination of factors, including family history, certain medications, substance abuse, chronic health problems, and major life changes [2,3]. Depression has an overwhelming influence on public mental health worldwide, affecting about two percent of the world’s population in 2017 [4]. People with depression show signs of sadness, difficulty concentrating, loss of appetite, loss of interest, impaired sleep, and, in the worst cases, self-denial leading to suicidal behavior. There are the preferred antidepression drugs, including selective serotonin reuptake inhibitors (SSRIs), tricyclic antidepressant amitriptyline, and monoamine oxidase inhibitors [5]. However, the widely used antidepressant treatment regimen has various disadvantages, including drug tolerance, delayed action, unsatisfactory efficacy, and various adverse reactions [6]. Therefore, finding new potential therapeutic measures for depression is significant, and the gut microbiota might provide insights.

As technology has progressed, more attention has gradually been paid to exploring the application of the intestinal microbiome as a therapeutic target for diseases. Recent research has indicated that the gut microbiota has shown great therapeutic potential in diseases such as gastrointestinal cancer, type II diabetes, autisms, Alzheimer’s, and Parkinson’s disease [7,8,9,10,11]. In terms of mental illness, there are numerous studies showing a strong association with mental illness and gut microbiota, including depression.

Recently, an increasing number of studies have pointed out that depressed patients show intestinal microbiota disorder accompanied by irritable bowel syndrome (IBS) [12]. Through high-throughput sequencing of depressed patients’ feces and comparison with that of healthy people, it was found that the microbial composition in depressed patients has changed [13]. The researchers also used fecal microbiota transplantation (FMT) to show that modification in the gut microbiota can cause or alleviate depression [14]. This bidirectional linkage between the intestinal microbiota and central nervous system (CNS) in patients with depression demonstrated that influencing gut microbiota might be beneficial in the treatment of depression [14,15,16]. Based on these findings, the effect of gut microbes in depression alleviating regimes and antidepression treatment has been firmly established, and a growing number of studies are devoted to the potential of applying intestinal microbes in the adjuvant therapy of depression.

This article critically reviews the current literature about the potential mechanism association between the microbiota–gut–brain axis and depression, and explores the current evidence of the feasibility of the intestinal microbiota in the relief of depression symptoms. In this review, we summarize the linkage between the intestinal microbiota and depression, and integrate evidence further to discuss the possible contribution of the intestinal microbiota to the antidepression effect.

## 2. Depression Mechanism and Existing Treatment Options

Depression is classified as a common mood disorder which, according to a global survey, occurs in more than 264 million people regardless of gender or age [17]. Depression often occurs when people are suffering a stressful life or devastating change. Indeed, this kind of intractable mental disease has various negative effect symptoms, including feelings of sadness, loss of interest, memory loss, and self-deprecation; it also has bad effects on the normal life of the patient and those around them [18].

The contributing factors of depression are complex and are often thought to be related to biological, psychological, and social factors, even including childhood trauma [19,20,21,22,23]. Studies focused on the physiological mechanism level indicate that depression might be related to low levels of brain-derived neurotrophic factor (BNDF), abnormal function of the hypothalamic–pituitary–adrenal axis (HPA), and glutamate-mediated toxicity, and may even be a result of structural and functional brain abnormality [24,25,26]. Furthermore, the mechanism of depression is also explained by the serotonin system, corticotrophin system, and dopamine system [26,27,28]. Chronic stress and severe depression often lead to further structural changes in the brain, usually reflected in hippocampus volume changes, neurogenesis, and neuronal apoptosis [29,30]. The hippocampus is connected to nerve fibers in areas of the brain associated with emotion, such as the prefrontal cortex and amygdala, and is involved in regulating pressure on the HPA axis by secreting nerve factors [31].

By analyzing the existing literature on the mechanism of depression, we can find that stress is a crucial intermediate factor of external factors acting on the body to produce depression. The mechanism of stress is based on the serotonin system, hypothalamus–pituitary axis, corticotropin releasing factor, and dopamine system [5,32]. These factors interact with each other to aggravate depression; for example, chronic stress and endocrine disruption caused by disruption form positive feedback, leading to depression [32,33,34].

Existing treatments for depression mainly rely on pharmacotherapy. The most used first-line drug is serotonin reuptake inhibitors (SSRIs); serotonin-norepinephrine reuptake inhibitors (SNRIs) and monoamine oxidase inhibitors are also used. Although existing antidepressant drugs have some efficacy, they are very limited, and in clinical cases, they can only provide alleviation and prevention [35]. Moreover, tolerance has been found in subsequent treatment—the same drug being used more than once in the same patient was less effective. Up to 35 percent of people are known to have treatment-resistant depression (TRD), the symptoms of which cannot relieved after four or more conventional treatments [36]. For the sake of TRD patients and ensuring the quality of life of all depression patients, it is indispensable to explore new antidepressant methods.

## 3. The Crucial Role the Gut Microbiota and Gut–Brain Axis Play in Central Nervous System Diseases

Microbiota can be found all around body surfaces and cavities [37]. The gut microbiota is described as the community of microbiota which colonize the intestinal tract and participate in a variety of physiological activities of the host [38]. These intestinal microorganisms get involved in various basic activities of host life, including nutrition metabolism, maintaining the structure of the intestinal mucosal barrier, immune regulation, and resistance to pathogens [39].

Based on the bidirectional regulation of the gut microbiota and the body, scientists regard the intestinal microbiome as a virtual organ in the human body which interacts with the human body and takes part in the metabolism and regulation of life activities [40]. The dynamic composition of the gut microbiota is regulated by various factors, including nutrition, lifestyle, hormonal changes, immunity, health situation, the application of drugs, and possibly microbiome–gut–brain axis crosstalk (e.g., stress, depression, autism) [41,42]. On the contrary, intestinal microorganisms can act on the host and regulate the host body by metabolites [43]. The gut microbiota can function as a rich repository of information, which is 100 trillion microbes in the gut, but only about 40 to 60 trillion cells in the human body [44], and the gene set of the intestinal microbiota is 150 times that of the human gene complement [45].

The dynamic structure and composition of the gastrointestinal microbiota are a mutual selection between the microorganism and the host, which improve interconnecting cooperation and functional stability within this complex ecosystem. Fluctuations in the gut microbiota within a certain range of limitation are considered as physiological health conditions. However, severe disruptions of the gastrointestinal microbiota will result in disease such as appetite loss, diarrhea, malnutrition, vitamin deficiency, and other symptoms, and even serious cases such as spontaneous bacterial peritonitis, hepatorenal syndrome, hepatopulmonary syndrome, and hepatic encephalopathy [46]. Nowadays, it has been indicated that the gastrointestinal microbiota can have an effect on central nervous system disease, including autism, depression, Parkinson’s disease, and Alzheimer’s disease, which is able to be illustrated by the gut–brain axis.

The gut–brain axis is an integrated physiological concept involving afferent and efferent nervous, endocrine, and immune signals between the central nervous system and the gastrointestinal system [27,47]. The conception of the gut–brain axis was primarily carried out by studying the connection of digestive function and satiety [15]. However, this concept has now been developed into the description of the interaction between the central nervous system and gastrointestinal system—especially the gut microbiome. The communication progress is regarded as bidirectional; therefore, the gut can regulate emotional activity in the CNS, and the CNS can regulate gastrointestinal function in turn [48,49].

The gut–brain axis is the interaction of the central nervous system, the enteric nervous system, and the digestive system [50]. The intestinal microbiota interacts bidirectionally with major parts of the central nervous system through a variety of direct and indirect pathways. Based on the literature to date, intestinal microorganisms are involved in basic physiological processes, including digestion, growth, and immune defense [48,51,52]. In addition, gut microbes, the enteric plexus, and the central nervous system also regulate responses to each other [47]. The nervous system affects the composition of the intestinal microbiota by modulating gut motility, the secretion of hormones, and the production of acid, bicarbonates, and mucus [52]. The secretions of intestinal microorganisms can enter the systemic circulation through the small intestinal epithelium, and some of them can directly act on the brain through the blood–brain barrier [53].

## 4. The Gut Microbiota and Microbes Interact Bidirectionally in People with Depression

### 4.1. Variation Occurs in the Intestinal Microbiota of Depression Patients

Depression patients commonly suffer from gut–brain dysfunction as well, such as appetite disturbances, metabolic disturbances, functional gastrointestinal disorders, and gut microbiota abnormalities [54,55]. According to the analysis of existing studies, we are able to conclude that the gut microbiota of depressed people is significantly different from that of healthy people. Studies commonly collect feces samples from depression mouse models or humans and analyze the microbiota species by high throughput molecular sequencing technology. Generally, the human gut microbiota consists of five phyla: *Bacteroidetes, Firmicutes, Actinobacteria, Fusobacteria*, and *Protobacteria*. Among them, *Bacteroidetes* and *Firmicutes* are the predominant phyla. These two phyla also appear to be the most affected in depression according to the literature [56,57]. Across all five phyla, nine bacteria were found to have a larger population in depression (*Anaerostipes, Blautia, Clostridium, Klebsiella, Lachnospiraceae incertae sedis, Parabacteroides, Parasutterella, Phascolarctobacterium*, and *Streptococcus*), six had a smaller population (*Bifidobacterium, Dialister, Escherichia/Shigella, Faecalibacterium*, and *Ruminococcus*), and six were conflicting (*Alistipes, Bacteroides, Megamonas, Oscillibacter, Prevotella*, and *Roseburia*) [13]. According to the variation of intestinal microbiota in patients with depression, we can demonstrate that the central nervous system impairment caused by depression will have an impact on the intestinal tract. Disorders of the gut microbiota caused by depression can lead to gastrointestinal symptoms, the most prevalent of which is irritable bowel syndrome (IBS)—both are accompanied by the increase of *Bacteroidetes* and *Firmicutes* [58]. Moreover, researchers have revealed that patients accompanied by IBS carry a higher risk of getting depression, and that disturbed intestinal microbiota is more common in these patients [59].

### 4.2. Intestinal Microbiota Can Regulate CNS in Depression Patients

Through experiments, it was found that the changes of intestinal microbiota in the central nervous system of patients with depression are not unidirectional: the intestinal microbiota can alleviate the symptoms of the central nervous system as well. An experiment carried by Kelly et al. showed that transplanting feces from depression patients into mice could generate depression-like behaviors [37]. In another experiment, researchers’ detection of interferon γ (IFN-γ) and the tumor necrosis factor-alpha (TNF-α) in the hippocampus increased in the brains of the mice which received the microbiota [16]. The recipient mice showed the same depression activities as their donor, including a loss of happiness, increased anxiety activity, and tryptophan metabolism dysfunction. Zheng harvested microorganisms from the excrement of depression patients (experimental group) and healthy people (control group), then transplanted them into germ-free mice. As a result, they found that the experimental mice exhibited higher depressive symptoms, and their microbiota composition was different from that of the control mice [14]. These experiments prove that composition variation in the intestinal microbiota can react on the nervous system and result in depression [37]. Sun et al. demonstrated that *L. plantarum* WLPL04 treatment can relieve host anxiety behavior [60]. The gut microbiota of patients with alcoholism can cause their hosts to exhibit depressive and anxious behaviors, and these changes can be spread through fecal bacteria transplants [61,62].

After further study, it was found that the mechanism of action of intestinal microorganisms may be related to their secretion of metabolites’ metabolism molecules [43]. First of all, the intestinal microbiota has a perfect structural basis for easy communication with the host, for which its secretions can communicate with the host through only one layer of small intestinal epithelial cells [63]. The molecules move across the intestinal epidermis and enter the inner circulatory system to act on the nervous system. LPS expressed by Gram-negative bacteria can affect central nervous system activity through mechanisms that have been shown to be related to the immune system and vagus nerve [64]. Microbial metabolites such as short-chain fatty acids and microbial neural particles such as catecholamine, histamine, and gamma-aminobutyric acid (GABA) may directly or indirectly affect the brain [13]. Gut microbes may also control serotonin levels by regulating tryptophan metabolism [65]. Short-chain fatty acids are also thought to be an important influence on bidirectional communication between the gut and brain [63].

In addition to the direct factors between intestinal microbes and the central nervous system, some external factors can act as intermediate factors and simultaneously affect both. The stress response in particular is considered as an important intermediate factor of depression which can be explained by the gut–brain axis. The intestinal changes caused by stress usually include two mechanisms: changes in intestinal motility dynamics and changes in intestinal barrier permeability [66,67]. In a 2013 study by Park et al., the researchers used bilateral olfactory bulbectomy mice as a model of stress-induced depression for study [68]. The results showed that the hypothalamic–pituitary–adrenal (HPA) axis was activated and the intestinal motility changed with the increased concentration of corticotropin-releasing hormone (CRH) [68]. These changes in intestinal motility and permeability were accompanied by transformation in the composition and stability of the gut microbiota after subsequent examination. Moreover, by measuring the prevalence and median values of serum IgM and IgA against lipopolysaccharide (LPS) in patients with depression, which have a negative effect on depressed patients, we found that the intestinal permeability of inflammatory factors was stronger in patients with depression [69].

The most common disease caused by stress-induced gut microbiota disorder is irritable bowel syndrome (IBS). IBS is a kind of general functional gastrointestinal disorder which is characterized by chronic and periodic unpleasant abdominal symptoms [70], which is considered related to variation of the gut microbiota system, epithelial barrier functions, and inflammatory response [39]. These intestinal symptoms include visceral pain, which travels to the central nervous system through the C nerve fiber and can be accompanied by negative emotions, which can create stress and worsen depression and form positive feedback [71].

**Figure 1:**
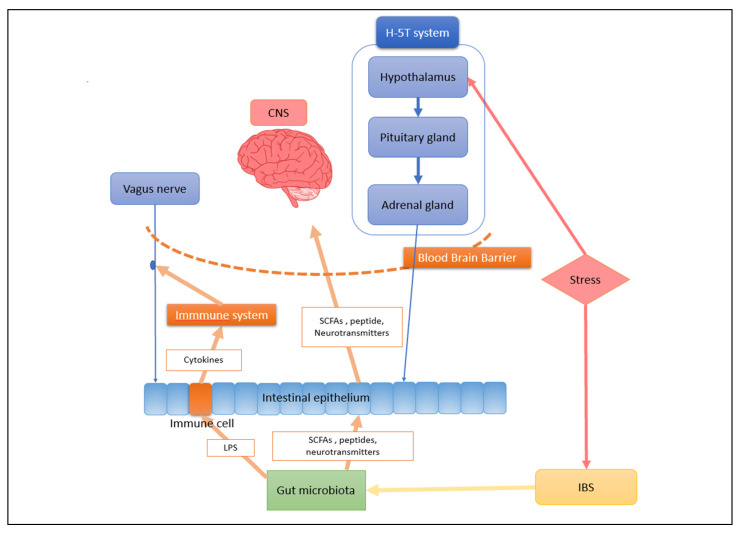
The communication pathway of the gut–brain axis in a depression patient. The HPA axis is activated under internal or external pressure [72]. The hypothalamus activates the release of CRH, promotes the release of ACTH by the pituitary gland, and results in the release of cortisol by the adrenal gland [72]. Cortisol is a hormone that affects intestinal integrity, motility, and mucus production, inducing changes in the composition of intestinal microbiota. In patients with depression, this pathway is significantly enhanced and HPA axis activity is overactive [73]. The end result is often IBS and disrupted gut microbiota. Microbial metabolites including short-chain fatty acids and microbial neural substrates such as catecholamines, histamines, and GABA can act on intestinal epithelial cells and stimulate intestinal nerves, which will stimulate the central nervous system through the vagus nerve [74]. Some SCFAs and peptide can cross the blood–brain barrier and act directly on the central nervous system. LPS on the surface of Gram-negative bacteria can mediate the effects of immune cells and the vagus nerve on the brain [75]. HPA, hypothalamus–pituitary–adrenal; CRH, corticotropin-releasing hormone; ACTH, adrenocorticotropic hormone; CNS, central nervous system; GABA, γ-aminobutyric acid; SCFA, short-chain fatty acid; LPS, lipopolysaccharide; IBS, irritable bowel syndrome.

The further mechanism of the communication between the gut microbiota and CNS has been illustrated in Figure 1.

## 5. Treatments of Depression

### 5.1. Traditional Treatment

Traditional treatments for depression include psychotherapy, medication, and electric shock therapy. The choice of treatment depends on the severity of the disease. In milder cases, exercise has been demonstrated to be a very effective treatment [76,77]. Usually, patients with mild symptoms are treated with psychotherapy and medication. Emergency electroshock therapy is used in very serious life-threatening cases, especially in cases with suicidal intention [78]. The most common type of drug treatment is with selective serotonin reuptake inhibitors (SSRIs) [28]. However, the limitations of SSRIs are obvious, as the responses are delayed and vary from patient to patient. In addition, brain-derived neurotrophic factor (BDNF) is also an important target for depression treatment based on the BNDF hypothesis [79]. It is believed that the occurrence of depression is related to the decrease of BNDF and its resulting neuronal apoptosis [80]. Therefore, there is a kind of antidepressant (e.g., fluoxetine) aimed at reducing hippocampal neuronal apoptosis by increasing the neurotrophic factor and nerve regeneration, so as to achieve the goal of improving patients’ depressive symptoms [81,82]. In addition, chronic central neuroendocrine abnormalities in patients with depression can induce structural changes in the subgenual cingulate region and become overactive; they are drug resistant and are usually repaired by electrical stimulation of the subgenual cingulate white matter [83,84].

Recent research has found that traditional treatments not only act directly on the nervous system, but also alter the structure of the gut microbiota system. For example, serotonin, a traditional antidepressant, also improves the gut microbiome while treating depression [85]. Even Qu and his colleagues found that the use of traditional Chinese medicine for depression was associated with an improvement in the gut microbiota [86]. Therefore, we can reason that their antidepressant effect might be partially connected with the adjustment of the microbe–gut–brain axis. Other therapies also target modifying gut microbiota composition. In addition, it has been demonstrated that the metabolic products of intestinal microorganisms have synergistic effects on antidepressant effects and pharmacokinetics [87]. Newer antidepressants even target regulation of the gut microbiota in the hopes of improving the disease. Yan et al. demonstrated that a polysaccharide (OP) which was isolated from okra (Abelmoschus esculentus (L) Moench), used in the treatment of the microbial–enteric–brain axis, achieved a good therapeutic effect [88].

### 5.2. Diet and Prebiotics

Diet is a crucial factor affecting the composition of intestinal microbes. The effect of food on the gut microbiota is significant, and research has revealed that dietary changes within a limited time (24 h) can apparently improve the structure of the intestinal microbiota system [89,90]. This suggests that we can harness the significant impact of diet on gut microbes to improve them, which could be presented as an option for adjuvant antidepression therapy. It has been proven that some component of diet can keep the structural homeostasis of the gut microbiota, and this effect is considered to have the potential to be put into clinical application. Through dietary relief, patients can reduce the psychological resistance to treatment to alleviate the effect of mild symptoms.

Prebiotics, one of the most commonly used dietary supplements, are non-digestive fibers which are partially digested in the gastrointestinal tract and support the growth of helpful gut bacteria [74]. Prebiotics, including galacto-oligosaccharide (GOS) and fructo-oligosaccharide (FOS), insulins, and oligofructose, in the diet may regulate the ecosystem structure of the intestinal microbiota system, which is significantly decreased in depression patients, such as *Lactobacillus*, *Bacteroides*, and *Bifidobacterium* [91,92]. In 2015, Andrew’s experiment demonstrated that the oligosaccharides 3′sialyllactose (3′SL) or 6′sialyllactose (6′SL) in human breast milk have the effect of restraining the development of anxiety [93]. In 2019, Richard et al. collected multiple clinical samples and data to study the efficacy of prebiotics on depression with meta-analysis. People generally support the antidepressant and anti-anxiety effects of probiotics, but due to the inaccuracy and insufficient number of clinical tables, further validation is still lacking [94].

In 2016, Schnorr and Bachner treated an anxiety patient with a combination of diet and psychotherapy. They eliminated diets that lead to hyperglycemia and added probiotic-rich foods to their diets. The results showed that the comprehensive antidepressant therapeutic regimen diminished the anxiety and insomnia symptoms of patients, increased the amount of helpful microorganisms such as *Lactobacillus*, decreased the abundance of harmful microorganism such as *Clostridium*, and changed the composition and diversity of bacteria [95].

### 5.3. Psychobiotics

Previous studies have shown that an abnormal intestinal microbiota leads to a decline in the stability of the gastrointestinal barrier, resulting in an increase of LPS entering the body, which activates systemic inflammation and promotes the stress response [96]. However, the treatment of probiotics can effectively prevent intestinal barrier damage and work through multiple mechanisms of action to reduce the response of the HPA axis to stress [97,98,99]. *Lactobacillus rhamnosus* probiotics can regulate the excitation-induced plasma corticosterone level and relieve depression through hormones such as BNDF and oxytocin secreted by the vagus nerve and hippocampus [100,101,102]. The mechanism of action of *Bifidobacterium* *infantis* is related to hormones such as acetylcholine and corticosterone and vagal nerve regulation [103,104]. In addition, when mixed strains probiotic supplement preparation (PSP) was used on anxious college students, anxiety was reduced in the experimental group in comparison with the control group [105].

Different kinds of probiotic may have different effects on different patients. A study finding suggests that a daily complement of *L. casei Strain Shirota* improves the composition of the intestinal microbiota, preserves its function, and may even alleviate symptoms of exposure to stress in healthy subjects [106]. However, another study showed that when all subjects were healthy, the improvement by intaking *L. casei* was not significant [107]. The benefits of probiotics became apparent only when the subjects were stressed or depressed [107]. In another study, however, healthy participants who were fed a diet including *L*. *helveticus* and *B. longum* for 30 days showed that the intake of the strain benefited stress states and negative mood regulation [108].

Bamling et al. applied an innovative combined antidepressant treatment by incorporating probiotics, magnesium, and SSRIs in 2017, hoping to have an effect on treatment resistant depression (TRD). The results were good: patients had significant relief in depressive symptoms [109].

### 5.4. Engineered Bacteria

Since the interconnection mechanism between the intestinal microbiota and central nervous system was gradually revealed [66], it has been demonstrated that the mechanisms of interaction between the gut microbiome and nervous system include: serotonin and tryptophan metabolism, the immune system, gut hormonal response, and short-chain fatty acids from bacterial metabolites [110]. This may provide a new therapeutic idea to enable engineered strains to continuously express therapeutic metabolites after intestinal colonization, so as to achieve therapeutic effects [111]. *L-4-chlorokynurenine (L-4-Cl-KYn)* is a novel neurochemical antidepressant that is being used in the therapeutic regime of depression. Dr. Hanna in recent times discovered that marine bacteria express this sequence, and, combined with the natural enzymes in the strain, will better exhibit its curative effect [112]. Targeting intestinal peptide release may be a way to combine with future breakthroughs in psychology. It can concentrate on peptide-mediated immune responses, promote vagal signaling, or regulate neuropeptide expression in certain brain areas to achieve the goal of symptom relief.

However, intestinal peptides have many limitations in practical application, such as a short half-life and blood–brain barrier crossing rate [113,114]. To solve this problem, using engineered strains with a targeted modification of peptide chemistry, intestinal peptides can be more effective. Better sequences of modified peptide chains and selection of strains are still being explored.

### 5.5. Fecal Microbiota Transplantation (FMT)

FMT is a method of transferring fecal bacteria from a donor to a recipient and is widely applied in treatment and research. Many studies have demonstrated that anxiety and depressive behaviors can induce depressive behaviors and stress responses in healthy hosts through fecal bacteria transplantation [16,61,115]. Although FMT has been used in scientific research on depression, it is rarely used with the therapeutic effect of psychiatric disorders. Until 2017, Kang et al. applied FMT to relieve autism and continued to improve both gastrointestinal and autism symptoms [116].

Under stress and depression, the gut microbiota can become disorganized, having a negative effect on the central nervous system and worsening the disease. However, FMT has shown outstanding results in the treatment of microbial structural disorders [117] and depression [118,119], also reprogramming the host’s metabolism [120]. Geng et al. have announced that FMT can regulate the diversity and ecosystem structure of colon microbiota, reduce the intestinal epithelial validation response, and reduce the host inflammatory response. Serotonin and 5-HT levels in the host are regulated [121]. Zhang et al. demonstrated that the transplantation of *NLRP3 KO* flora alleviates anxiety behavior in wild-type mice [122].

IBS is a common complication of depression, and the treatment of IBS and alleviation of depression may have therapeutic effects on both diseases [123]. In the treatment of IBS, FMT has been included in common treatment plans as a treatment with significant efficacy [124,125]. The remission rate of IBS patients who finish FMT treatment can be up to 89% [126]. Therefore, the treatment of FMT in patients with depression combined with IBS can be a focus of research.

The current treatment of fecal bacteria transplantation usually adopts endoscope, enema, and orally taken freeze-dried materials to achieve the effect of transplantation of bacteria [127,128]. Other types of transplants are being explored, such as Microbial Ecosystem Therapeutics-2 (MET-2). MET-2 is used to extract gut microbes from the stool samples of healthy people, and is then screened, cultured in a laboratory, and treated with industrial food before being given to patients [129,130]. Furthermore, FMT is considered to be a more effective and safe treatment. In the course of FMT treatment, pathological side effects were found due to changes in intestinal microbiota [131,132,133] and most of the adverse reactions in these studies were mild [132] or self-limited [131,133] (Table 1).

## 6. Conclusions and Outlook

With increasing pressure in modern life, depression has become a common disease, yet there is no complete treatment for the disease. According to the literature on the gut–brain axis, depression has a lot to do with changes in the gastrointestinal microbiota. Depression can alter the composition of the gut microbiota and even lead to a complication called IBS. Disturbances in the gastrointestinal microbiota can further cause stress responses and worsen depression. Disruption of the gut microbiome can lead to IBS, which increases the risk of depression. The purpose of relieving the depressive symptoms of patients can be achieved by adjusting and modifying the composition of microorganisms. Therefore, we concluded with the inductive integration of the changes in the intestinal microbiota of patients with depression and research about microbiota targeting measures.

Existing alleviating regimens and antidepressant treatment associated with gut microbes include traditional microbial-targeting drugs, dietary therapy, prebiotics, probiotics, engineered bacteria that express antidepressants, and fecal microbiota transplantation. Acceptable outcomes can be achieved in the prevention and remission of depression by regulating the intestinal microbiota and utilizing the mechanism of action of the gut–brain axis. Moreover, these measures are more flexible and maneuverable, which allows patients to use non-drug measures to relieve symptoms and improve their quality of life.

According to the molecular mechanism and bacterial species changes of depression summarized in this paper, the future research direction can be based on the adjustment of the bacterial community structure in patients with depression, or the synthesis of target molecules using engineered bacteria.

## Figures and Tables

**Table 1:** Effects of microbiological treatments on mouse models and human behavior.

Author	Treatment	Experimental Subjects	Main Results
Yan et al. [88]	A polysaccharide (OP) which is isolated from okra (Abelmoschus esculentus (L) Moench)	CUMS-induced mice and fecal microbiological transplantation (FMT)-induced mice were used as models of depression	OP can treat depression through the microbial–gut–brain axis
Vulevic et al. [92]	Prebiotics-FOS1 and GOS	C57BL/6J male mice	Chronic prebiotics FOS1 GOS showed antidepressant and anti-anxiety effects
Tarr et al. [93]	Oligosaccharides 3′sialyllactose (3′SL) or 6′sialyllactose (6′SL)	6~8-week-old male C57/BL6 mice used of the social disruption stressor	This prebiotic has a preventive effect on anxious behavior and inhibits nervous anxiety-related responses
Benton et al. [107]	Milk with probiotics	124 healthy members of general population	There was an improvement in subjects, a positive effect on mood, and probiotic intake was also associated with demonstrated memory
Qin et al. [105]	Probiotic supplement preparation (PSP)	120 college students with anxiety trend	Anxiety parameters decreased in the experimental group compared to the control group
Bravo et al. [100]	*Probiotic Lactobacillus rhamnosus (JB-1)* administration	Stress induced anxiety- and depression-like behaviors mice	Reduced stress-induced anxiety- and depression-like behaviors were found in mice
Messaoudi et al. [108]	*Lactobacillus casei strain Shirota*	Subjects’ mild depressive symptoms and low scores	Long-term combined use of *Lactobacillus helveticus R0052* and *Bifidobacterium longum R0175* reduced the symptoms of people with mild depression and effectively alleviated anxiety
Kang et al. [116]	Human fecal extract and FMT	Adult germ-free (GF), Swiss Webster female mice	Fecal bacteria transplantation can change the neural structure of the brain and affect the brain
Geng et al. [121]	FMT	Newborn piglets and subsequent lipopolysaccharide (LPS) challenge	FMT can regulate tryptophan metabolism and improve intestinal microbial disorders
Rao et al. [119]	FMT	Mice with chronic depression-like behavior induced by mild external stressors	FMT improves stress-induced depression-like behavior associated with inhibition of rat brain glial cells and NLRP3 inflammasome
Zhang et al. [122]	Transplantation of the NLRP3 KO microbiota from NLRP3 KO mice	NLRP3 KO mice	FMT from NLRP3 KO mice significantly alleviates the depressive-like behavior induced by chronic unpredictable stress (CUS)-induced depressive-like behaviors
